# Combined Long-Term Androgen Deprivation and Pelvic Radiotherapy in the Post-operative Management of Pathologically Defined High-Risk Prostate Cancer Patients: Results of the Prospective Phase II McGill 0913 Study

**DOI:** 10.3389/fonc.2020.00312

**Published:** 2020-03-12

**Authors:** Michael Jonathan Kucharczyk, James Man Git Tsui, Farzin Khosrow-Khavar, Boris Bahoric, Luis Souhami, Maurice Anidjar, Stephan Probst, Ahmad Chaddad, Paul Sargos, Tamim Niazi

**Affiliations:** ^1^Department of Radiation Oncology and Radiation Physics, Nova Scotia Cancer Centre, Dalhousie University, Halifax, NS, Canada; ^2^Department of Radiation Oncology, Jewish General Hospital, Montreal, QC, Canada; ^3^Department of Radiation Oncology, McGill University Health Centre, Montreal, QC, Canada; ^4^Department of Oncology, McGill University, Montreal, QC, Canada; ^5^Department of Urology, Jewish General Hospital, Montreal, QC, Canada; ^6^Department of Nuclear Medicine, Jewish General Hospital, Montreal, QC, Canada; ^7^School of Artificial Intelligence, GUET, Guilin, China; ^8^Département de radiothérapie, Institut Bergonie, Bordeaux, France

**Keywords:** radiotherapy, adjuvant, salvage, androgen deprivation therapy (ADT), pelvic lymph node radiotherapy, prostate cancer, clinical trial

## Abstract

**Purpose:** Following radical prostatectomy, prostate bed radiotherapy (PBRT) has been combined with either long-term androgen deprivation therapy (LT-ADT) or short-term ADT with pelvic lymph node radiotherapy (PLNRT) to provide an oncological benefit in randomized trials. McGill 0913 was designed to characterize the efficacy of combining PBRT, PLNRT, and LT-ADT. It is the first study to do so prospectively.

**Methods:** In a single arm phase II trial conduced from 2010 to 2016, 46 post-prostatectomy prostate cancer patients at a high-risk for relapse (pathological Gleason 8+ or T3) were assessed for treatment with combined LT-ADT (24 months), PBRT, and PLNRT. Patients received PLNRT and PBRT (44 Gy in 22 fractions) followed by a PBRT boost (22 Gy in 11 fractions). The primary endpoint was progression-free survival (PFS). Toxicity and quality of life (QoL) were evaluated using CTCAE V3.0 and EQ-5D-3L questionnaires, respectively.

**Results:** Among the 43 patients were treated as per protocol, median PSA was 0.30 μg/L. On surgical pathology, 51% had positive margins, 40% had Gleason 8+ disease, 42% had seminal vesicle involvement, and 19% had lymph node involvement. At a median follow-up of 5.2 years, there were no deaths or clinical progression. At 5 years, PFS was 78.0% (95% Confidence Interval 63.7–95.5%). Not including erectile dysfunction, patients experienced: 14% grade 2 endocrine toxicity while on ADT, one incident of long-term gynecomastia, 5% grade 2 acute urinary toxicity, 5% grade 2 late Urinary toxicity, and 24% long-term hypogonadism. No comparison between the average or minimum self-reported QoL at baseline, during ADT, nor after ADT demonstrated a statistically significant difference.

**Conclusions:** Combining PBRT, PLNRT, and LT-ADT had an acceptable PFS in patients with significant post-operative risk factors for recurrence. While therapy was well-tolerated, long-term hypogonadism was a substantial risk. Further investigations are needed to determine if this combination is beneficial.

**Trial registration:** NCT01255891.

## Introduction

Three large randomized phase III trials of salvage radiotherapy in prostate cancer have investigated combining prostate bed radiotherapy (PBRT) with androgen deprivation therapy (ADT) and/or pelvic lymph node radiotherapy (PLNRT). GETUG-AFU 16's salvage patients had a benefit in metastasis-free survival when short-term ADT (ST-ADT) was added to locoregional treatment (PBRT with PLNRT or previous dissection), compared to no ADT (1). RTOG 0534, also called SPPORT, noted improvements in biochemical progression-free survival (PFS) by adding PLNRT to the combination of PBRT and ST-ADT, compared to PBRT and ST-ADT only (2). RTOG 9601 has been the only trial to show a survival advantage when long-term ADT (LT-ADT) was added to PBRT, in comparison to PBRT alone, in salvage patients at a higher risk for recurrence albeit with substantially longer follow-up (3).

To date, no trial of salvage radiotherapy has incorporated both LT-ADT and PLNRT among an entire treatment arm. This approach has merit based on the survival benefit of LT-ADT over ST-ADT in high-risk localized prostate cancer patients receiving definitive external beam radiotherapy to the prostate and PLNRT (4). Given the poor prognosis in the setting of a clinical recurrence or with pathological node involvement post-prostatectomy, there may be a role for such a combination in adjuvant radiotherapy patients with significant risk factors for recurrence as well (5–9). McGill 0913 is the first prospective clinical trial of salvage radiotherapy for prostate cancer to report on combining PNRT and LTADT to PBRT. This paper reports on the PFS and quality of life (QoL) after 5 years of median follow-up for this surgically staged high risk population.

## Materials and Methods

### Patient Population

Patients with prostate cancer who underwent radical prostatectomy, with or without lymph node dissection, were approached for enrolment on a prospective basis. Eligibility criteria included at least one of the following: pre-prostatectomy prostate specific antigen (PSA) ≥20 μg/L, pathological Gleason score ≥8, or pathological T3 disease. Post-operative PSA levels were not an eligibility criterion. Patients were ineligible for any of the following: Karnofsky Performance Score (KPS) <70, age <18, inadequate marrow function (platelets <100,000 cells/mm^3^, hemoglobin <10.0 g/dL, or AST/ALT ≥2x the upper limit of normal), post-operative PSA ≥5.0 μg/L, pelvic lymphadenopathy ≥1.5 cm or distant metastases on imaging, residual tumor detectable on exam or imaging; prior pelvic radiotherapy, malignancy within the past 5 years, or an active severe co-morbidity.

### Treatment

ADT began 2–10 days after patient enrolment. All patients received 28 days of bicalutamide (50 mg once daily) and luteinizing hormone-releasing hormone (LHRH) agonist began [intramuscular injection of 22.5 mg of Lupron (leuprolide acetate) depot every 3 months] on day 15. Radiotherapy started 2 months following ADT's initiation.

Radiotherapy was delivered with photon energies of at least 6 MV using either a 3D conformal radiotherapy (prior to 2012) or intensity modulated therapy technique thereafter. Treatment consisted of pelvic radiation (phase 1) with 44 Gy in 22 fractions followed by a boost (phase 2) of 22 Gy in 11 fractions to the prostate bed. The clinical target volume of the first phase of radiotherapy included the prostate bed, original location and or remnants of the seminal vesicles, and draining lymphatics (internal iliac, external iliac, obturator, distal common iliac, and presacral lymph nodes) (10). Dose was prescribed to the isocenter for the 3D conformal technique and to the prespecified volume for the intensity modulated radiotherapy approach (100% of the prescribed dose to cover 95% of the volume).

### Assessments

Pre-treatment assessment included history, physical exam, questionnaires [EORTC QLQ-C30 (11), EQ-5D (12), International Index of Erectile Function (IIEF-5) (13), and International Prostate Symptom Score (IPSS) (14)], serological assessments (CBC, electrolytes, urea, liver function tests, testosterone levels, and PSA), chest x-ray, CT or MRI of the abdomen & pelvis, and a total body bone scan. During radiotherapy, patients were seen weekly by their Radiation Oncologist. Observed toxicities were reported using the Common Toxicity Criteria version 3 scale (CTCAEv3) (15). Following radiotherapy, patients were assessed every 3 months until 2 years, every 6 months for 3 years, and annually thereafter. At each follow-up visit, PSA, testosterone, EQ-5D, IPSS, IIEF-5, and toxicity were assessed. EORTC QLQ-C30 questionnaires were completed annually. If there was biochemical progression or symptomatic changes worrisome for progression, bone scan and CT of the abdomen and pelvis were repeated.

### Endpoints

The primary endpoint was PFS at 5 years. Progression included either biochemical failure or clinical progression. Prior to analysis, biochemical failure was defined as PSA exceeding nadir plus 0.2 μg/L and found to be elevated on two subsequent assessments. Clinical progression was defined as either a local or distant failure. A *post-hoc* reporting of the (1) primary endpoint among PSA subgroups (<0.34 and ≥0.34 μg/L) postulated to stratify the benefit PBRT treatment intensification and (2) of the PFS when biochemical failure was defined as nadir plus 2 μg/L were done to allow for an indirect comparison (2). Secondary endpoints included: (1) local failure rate at 5 years, (2) distant failure rate at 5 years, (3) toxicity rates, and (4) QoL. For the evaluation of testosterone recovery, the lower end of the normal range was considered 7 nmol/L.

### Statistical Analysis

The 5-year PFS with PBRT alone was estimated to be ~45% based on prior trial data (16). We designed this study with Fleming's one-stage method (17, 18). A 5 year biochemical and clinical progression free survival of 45% was considered ineffective for the combined treatment regimen with LT-ADT (24 months), PBRT, and PLNRT in this population whereas a 5 year progression-free survival of 65% or higher was considered to warrant further subsequent studies. Based on a binomial distribution with a one-sided type I error of 0.05, type II error of 0.15 (power of at least 85%), the study was designed to enroll 46 patients, with at least 26 patients required to be alive and progression free at 5 years to reject the null hypothesis in favor of alternative hypothesis (18).

Any PSA or testosterone level below the detectable threshold—typically <0.1 μg/L and <0.7 nmol/L, respectively—was set to 0 for the purpose of calculation. Castration was defined as a testosterone level <1.7 nmol/L while the patient was under the effect of ADT. Testosterone recovery was defined as the testosterone returning to the normal range (i.e., at least 7 nmol/L) and the end of ADT was considered to be 13 weeks following the final Lupron injection. Median follow-up was calculated from the first administration of Lupron injection. A Kaplan-Meier curve was constructed to obtain the biochemical PFS. We conducted a univariate analysis of baseline characteristics and testosterone levels at the time of treatment failure to investigate whether there was a statistical difference between groups of patients who failed vs. those who did not. Chi-square proportion test was used for binary data, otherwise Wilcoxon signed-rank test was employed.

## Results

### Participants

Patients were recruited from 2010 to 2016 to meet the accrual goal of 46 patients. Three patients were not treated as per protocol: one was found to be ineligible, one opted for surgery, and one refused radiotherapy. No patients had connective tissue disorders. On review of the remaining 43 patients, two did not meet the prospectively stated trial criteria—their post-operative PSA's were 5.7 and 9.8 μg/L at registration.

Significant pathological features included 19% (*n* = 8) with positive lymph nodes, 40% (*n* = 17) with Gleason ≥8, 63% (*n* = 27) with extracapsular extension, and 18 (*n* = 42%) with seminal vesicle involvement (Table 1). Before radiotherapy, the median PSA value was 0.30 μg/L [interquartile range (IQR)−0.20 to 0.47]. For 5 patients, the PSA was below 0.1 μg/L, 4 had PSAs between 0.1 and 0.19 μg/L, 6 had PSAs of 0.20 μg/L, 21 had PSAs between 0.21 and 0.50 μg/L, 3 had PSAs between 0.51 and 1.00 μg/L, and 4 had PSAs >1.00 μg/L. ADT was initiated within a median of 71 weeks post-surgery.

**Table 1:** Participants' baseline clinical and pathological features.

**Characteristic**		***n* = 43**
**Age**		
	Median (IQR)	65 (59–69)
**Pathological T stage**, ***n*** **(%)**		
	T1	2 (4.7%)
	T2	11 (25.6%)
	T3	30 (69.8%)
**Biopsy Gleason score**		
	7	26 (60.5%)
	8	12 (27.9%)
	9	5 (11.6%)
**Pathological Gleason score**		
	6	1 (2.3%)
	7	25 (58.1%)
	8	9 (20.9%)
	9	6 (14.0%)
	Unknown	2 (4.7%)
**Testosterone (nM/L)**		
	Median (IQR)	11.20 (8.28–16.10)
	Mean (SD)	12.31 (5.25)
	Unknown	2 (4.7%)
**Post-operative PSA**		
	Median (IQR)	0.30 (0.20–0.47)
**Margin status**		
	Negative	18 (41.9%)
	Positive	22 (51.2%)
	Unknown	3 (7.0%)
**Lymph node involved**		
	No	29 (67.4%)
	Yes	8 (18.6%)
	Unknown	6 (14.0%)
**Extracapsular extension**		
	No	9 (20.9%)
	Yes	27 (62.8%)
	Unknown	7 (16.3%)
**Seminal vesicle involvement**		
	No	23 (53.5%)
	Yes	18 (41.9%)
	Unknown	2 (4.7%)
**Time from surgery to post-operative therapy**		
	Median weeks (IQR)	68.3 (27.0–177.6)
**Duration of ADT prior to radiotherapy start**		
	Median weeks (IQR)	9.0 (8.0–10.9)

### Treatment Failure

**Figure 1:**
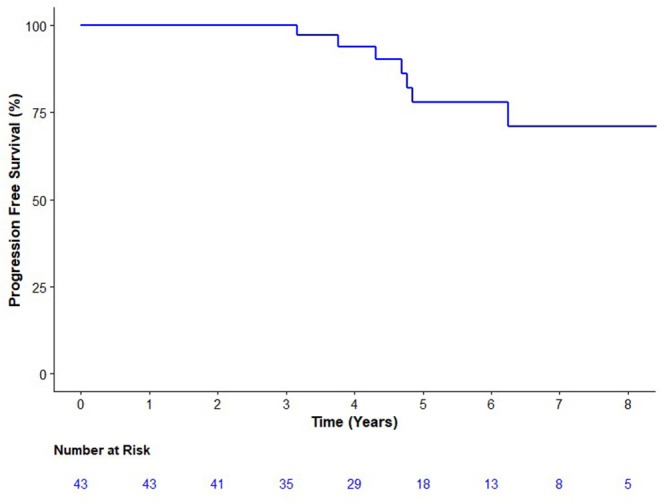
Kaplan Meier analysis of the progression-free survival. Progression was defined as either biochemical (prostate specific antigen nadir + 0.2 μg/L) or clinical progression on exam or imaging.

Seven patients have experienced biochemical failure at a median follow-up of 5.2 years (Figure 1). PFS at 3, 4, and 5 years were 100.0 [95% Confidence Interval (CI) 91.5–100%], 93.9 (95% CI 86.1–100%), and 78.0% (95% CI 63.7–95.5%), respectively, surpassing the predefined 5-year PFS of significance of 65%. There was one new failure after 5 years (at 6.2 years). None of the patients with biochemical failure had detectable disease on either examination or restaging investigations (i.e., no clinical failures). There have been no deaths among the study's population.

When treatment failure was defined as nadir plus 2 μg/L, the first failure was 4.8 years with a 5-year PFS of 92.1% (95% CI 82.2–100%). Among participants with a PSA below 0.34 ug/L, at 5 years there were two failures a PFS of 87.8% (95% CI 73.4–100.0). Among those with PSAs of at least 0.34 ug/L, at 5 years there were four failures and a PFS of 70.0% (49.2–99.7%). A test of interaction was not significant (*p* = 0.66).

During ADT, 11 patients had testosterone values that did not remain <0.7 nmol/L and 3 had values that did not remain <1.7 nmol/L. Castration levels of testosterone (<1.7 nmol/L) did not predict for progression in a univariate model (*p* = 0.43, Table 2). There were no biochemical or clinical failures in the 9 patients with baseline PSA values under 0.2 μg/L. Of the 8 node positive patients, 1 had subsequent biochemical failure. The remaining univariate analyses did not demonstrate any significant difference in biochemical failures according to baseline testosterone levels, pathological T stage, extent of prostatic involvement, Gleason score, PSA levels, lymph node involvement, extracapsular extension, seminal vesicle invasion, or perineural invasion.

**Table 2:** Adequate chemical castration (serum testosterone <1.7 nmol/L) compared to incidence of biochemical failure.

		**Castrate testosterone on ADT**		
		**Yes**	**No**	**Total**
Biochemical failure	Yes	7	0	7
	No	33	3	36

*In a univariate model, there was no statistical association with treatment failure (p = 0.43); ADT, androgen deprivation therapy; PSA, prostate specific antigen*.

### Toxicity

**Figure 2:**
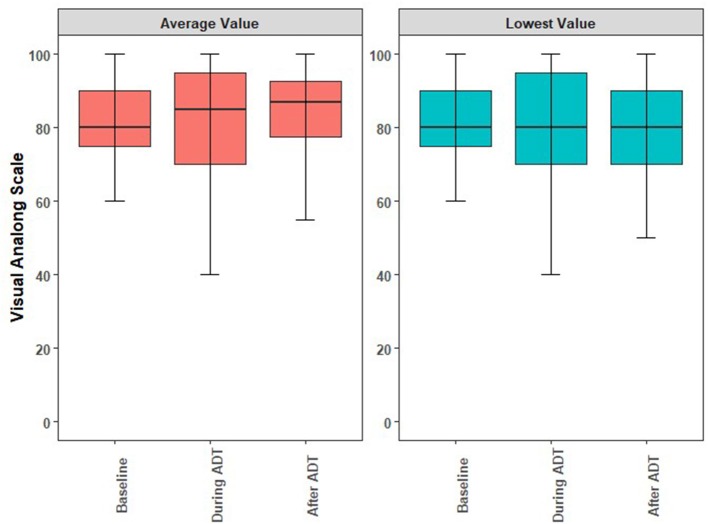
Boxplots illustrating the medians, interquartile ranges, and ranges of QoL reported by individual study participants at baseline, during ADT, and anytime following ADT. **(A)** Comparison of the averaged self-reported QoL. **(B)** Comparison of lowest self-reported QoL. ADT, androgen deprivation therapy; QoL, quality of life.

The acute and long-term adverse effects experienced by the patients are reported in Table 3. Excluding erectile dysfunction, no grade 3 toxicites were observed. Ten (23.3%) patients experienced a grade 2 toxicity. Regarding the overall QoL as assessed by the EQ-5D's visual analog score (12), there were no statistically significant changes observed while on or after ADT. While there was a decrease in QoL while patients were on ADT, this was not statistically significant (*p* = 0.39) and the mean differences are summarized in Figure 2. The mean minimum QoL experienced at any given time while on ADT by a patient was 7.8 (standard deviation = 2.0), compared to a mean baseline QoL of 8.2 (standard deviation = 1.2).

**Table 3:** Patients which experienced new grade 2 or greater toxicities, related to intervention.

	**During ADT (%)**	**Post-ADT (%)**
Grade 2 or greater ADT Induced Toxicity	5 (10.8%)	1 (2.2%)
	**Acute (<90 days)**	**Late (>90 days)**
Grade 2 or greater Radiation Induced Toxicity	2 (4.3%)	2 (4.3%)

*ADT induced toxicities included both endocrine and cardiovascular toxicities. All observed radiation induced toxicities were urinary; ADT, androgen deprivation therapy*.

Of the 9 (20.9%) patients who experienced any grade of gastrointestinal toxicity, 2 (4.7%) patients presented with rectal bleeding, one of which was diagnosed as radiation proctitis. Six patients (13%) experienced 8 instances of grade 2 or greater toxicities that were possibly associated with ADT. The toxicities were hot flashes (*n* = 2), and single instances of headaches, hypertension, fatigue, breast tenderness, long-term gynecomastia with breast tenderness, or stroke. The single patient experiencing stroke had this 8 months after initiating ADT. Hormonotherapy was thus stopped for this patient after a total of 3 injections.

At baseline, 21 patients (48.8%) reported some degree of urinary incontinence. There were 2 patients in the acute and 2 in the chronic period with grade 2 urinary genitourinary toxicities and there was no new chronic incontinence. The median IPSS scores at baseline, the first appointment following radiotherapy, and 2 years following the start of ADT were 6 (IQR = 2.0–9.3), 7.5 (IQR = 4.0–13.0), and 4.5 (IQR = 2.5–7.5), respectively.

Regarding erectile dysfunction, baseline function was reported as a median IIEF-5 of 5 (IQR = 5.0–9.5) and 28 men (65.1%) had already Grade 3 toxicity (decreased erectile function not improve with intervention). Among the men with a documented baseline IIEF-5 of 10 or greater, the median IIEF-5 at baseline and 3 years or more following the start of ADT was 15 and 8.5, respectively. Grade 3 erectile dysfunction was reported in all patients while ADT was ongoing.

The number of patients who completed 18 and 24 months of ADT were 27 (62.7%) and 19 (44.2%), respectively. Testosterone recovered within normal ranges within a year of ADT completion in 15 (34.9%) patients and 10 patients have not recovered their testosterone to normal levels. For the patients who achieved castration while on ADT, the median time to testosterone recovery was 78.0 weeks (IQR 51.7–104.0) after the last ADT injection.

## Discussion

McGill 0913 investigated the efficacy and toxicity of PBRT treatment escalation with PNRT and LTADT in patients with a pathological high risk of recurrence following prostatectomy. At its predefined endpoint of 5 years of median follow-up, 36 of 43 patients were alive and without disease progression, surpassing the predefined endpoint which would warrant further studies into this treatment regimen. To our knowledge, it is the only prospective study to report on this combination in this population.

The actual study population largely represented what would now be considered salvage radiotherapy patients, as opposed to adjuvant radiotherapy (19–22). McGill 0913's study population also included patients at a particularly high risk for subsequent disease progression, such as pathologically involved lymph nodes (7–9, 23). Despite this, there were few treatment failures (22%) at a median follow-up of over 5 years.

The most comparable trials are RTOG 9601 and SPPORT (2, 3). RTOG 9601 randomized node negative patients (*n* = 760) with a nodal dissection, PSAs between 0.2 and 4.0 μg/L (median 0.6 μg/L), and either pT3 disease or the combination of pT2 disease with a positive margin, to either PBRT alone or PBRT with LTADT (3). In contrast, SPPORT assessed a slightly lower risk population (*n* = 1,622) that excluded Gleason 9-10 disease, and randomized node negative T2-T3 patients with a PSA between 0.1 and 2.0 μg/L (median 0.34 μg/L) to either PBRT with STADT or PBRT with both STADT and PNRT (2). Regarding the populations, McGill 0913 participants' median PSA of 0.30 μg/L could suggest a relatively lower risk of recurrence (24). However, our heterogeneous population included participants with Gleason 9 disease, pathologically involved lymph nodes, and PSAs >2.0 μg/L could arguably place McGill 0913's cohort at a comparable or higher risk of recurrence.

RTOG 9601's addition of LT-ADT improved the 5-year biochemical failure from ~50 to 23% [Hazard Ratio (HR) = 0.48; 95% CI 0.40–0.58]. SPPORT's cohorts had 5-year freedom from progression improve from 71 to 87% with the addition of both STADT and PNRT to PBRT (HR = 0.45; 0.34–0.61). In addition to the limitations of indirect comparisons, this is further complicated by varying definitions of treatment failure (25, 26). McGill 0913's lower threshold for biochemical failure (nadir plus 0.2 μg/L) introduced a lead time bias. These patients may have been reported as failing at an earlier date (or not at all) if RTOG 9601's (nadir plus 0.3 μg/L) or SPPORT's (nadir plus 2.0 μg/L) definitions of biochemical failure were implemented instead. To facilitate indirect comparison, we performed an *ad hoc* analysis that showed a PFS of 92.1% at 5 years when biochemical failure was defined as nadir plus 2.0 μg/L. The rationale for the selection of these different endpoints was investigator discretion—defining biochemical failure with higher PSA thresholds was related to an increasing specificity and sensitivity to predict clinical failure, albeit at the cost of potentially delaying the next line of therapy (27).

Given the reported efficacy of multimodality salvage radiotherapy with either PNRT or LT-ADT added to PBRT, the toxicity of combining the therapies is highly relevant (2, 3). In the present study, the average changes in patient's overall QoL score while on ADT, compared to baseline values, were statistically insignificant. This observation is similar to other QoL assessments for LTADT (28) while adherence to ADT at 18 months was comparable between RTOG 9601 (69.8%) and McGill 0913 (63%). The low compliance for 24 months of ADT (44.2%) among McGill 0913 participants may be secondary to robust participation in PCS IV (29), a local randomized control trial finding that 18 months of ADT was non-inferior to 36 months among high risk prostate cancer patients. Following the publication of the RADICALS data, one subgroup of their 2 × 2 factorial design will have been treated with the combination of LT-ADT and PLNRT, at the investigators' discretion, and may provide future insight (19). At present, the McGill 0913 data offers prospective data this regimen appears to be well-tolerated by its participants.

While this study does imply potential efficacy of its treatment in a population that included patients at a great risk for recurrence, there are significant limitations. These include its single arm design, small sample size, a median follow-up that cannot yet reliably report on survival surrogates in prostate cancer, and heterogeneity in risk factors (e.g., a modest median PSA but numerous patients with involved nodes). What can be inferred is that multimodality treatment with PBRT, PNRT, and LTADT is well-tolerated, although it risks long-term hypogonadism.

Immintely reporting phase III clinical trials will address some of the uncertainties facing the management of the post-prostatectomy patients. The presented RAVES and RADICALS trials as well as the ARTISTIC meta-analysis suggest that salvage radiotherapy is of similar benefit to adjuvant radiotherapy, limiting the number of patients we may consider for adjuvant radiotherapy among patients with high-risk features for recurrence (30–32). GETUG-AFU 17 investigates a broader spectrum of patients at a greater risk for recurrence and compares adjuvant vs. early salvage radiotherapy, both combined with concurrent ST-ADT (20). Avenues for treatment intensification are being explored in two randomized trials—dose escalation in the maturing phase III SAKK 09/10 trial (33) and the addition of an androgen receptor axis therapy (ARAT) in an accruing phase II trial (34). Node positive patients are excluded from all five trials.

Given that ARATs have potentiated chemical castration in patients undergoing salvage radiotherapy (35), they may have a role in escalating therapy. The phase II STREAM trial treated locally advanced patients, including those with positive nodes. STREAM provided salvage radiotherapy with ST-ADT via enzalutamide alone (i.e., no LHRH-interacting agent) and compared their observed 2-year PFS of 65% to a historical standard of 51% (36). Two other awaited phase II studies evaluate the addition of either enzalutamide or apalutamide to salvage radiotherapy, though the one randomized study will only enroll pathologically node negative patients (34, 37).

The efficacy of treatments in patients with nodal involvement is less clear. McGill 0913's single treatment failure among 8 node positive patients is reassuring but inadequate to assess efficacy. Larger series (38) and a subpopulation analysis of STAMPEDE (39) have suggested benefit with pelvic radiotherapy in pathological or clinical node positive patients. More extensive analyses even suggest adjuvant radiotherapy and LT-ADT alone could suffice for patients with a limited nodal burden (40). However, these studies do not consider the previously occult nodal burden that may now be detected by increasingly implemented more sensitive modern functional imaging (41, 42). Ultimately, patients with involved lymph nodes are likely at an increased risk of recurrence, randomized evidence for their management is lacking, they may be detected more frequently, and the population is not included among the ongoing randomized trials (19–21, 34). A randomized trial incorporating ARATs and/or the combination of PBRT, PLNRT, and LT-ADT may be worth considering for this very high risk population.

## Conclusion

McGill 0913 is the only prospective clinical trial to investigate adding PNRT and LT-ADT to PBRT in the post-operative setting. The regimen appeared efficacious and tolerable in a cohort of surgically staged high risk patients, despite significant long-term hypogonadism. Given other randomized trials showing the tolerability of adding PNRT with LTADT, future trials could consider adding further treatment modalities for patients at a higher risk for recurrence.

## Data Availability Statement

The datasets generated for this study will not be made publicly available this was not included in the informed consent process—the patients consented to be studied, but not to have their clinical data shared. Requests to access the datasets should be directed to the corresponding author.

## Ethics Statement

The studies involving human participants were reviewed and approved by Research Ethics Board of the Jewish General Hospital. The patients/participants provided their written informed consent to participate in this study.

## Author Contributions

MK directed the analysis and interpretation of the data, composed the manuscript, and coordinated revisions. JT performed the analysis, assisted with data interpretation, and was greatly involved in manuscript revisions. FK-K validated the methods/data analysis, added additional statistical assessments, and provided manuscript revisions. TN and LS were involved in formulating the study. TN was the principal investigator, supervisor to MK, JT, and PS, and most heavily involved in guiding revisions. BB, MA, SP, LS, AC, and PS provided assistance with manuscript preparation, revisions. BB, MA, and LS assisted with the study's clinical undertaking.

### Conflict of Interest

The authors declare that this study received funding from AbbVie Pharmaceuticals.
